# Acute Hypoxemic Respiratory Failure Due to COVID-19 in a Patient With Underlying Lymphangioleiomyomatosis

**DOI:** 10.7759/cureus.25871

**Published:** 2022-06-12

**Authors:** Joseph Glowacki, Gregory Holland, Colleen Graham, Khazenay Bakhsh

**Affiliations:** 1 Internal Medicine, Rowan University School of Osteopathic Medicine, Stratford, USA; 2 Pulmonology, Jefferson Health New Jersey, Turnersville, USA

**Keywords:** sirolimus, mtor inhibitor, rare disease, acute hypoxemic respiratory failure, covid-19, lymphangioleiomyomatosis

## Abstract

Lymphangioleiomyomatosis (LAM) is a rare disorder that can cause lesions that develop into cysts, most commonly in the lung parenchyma and renal angiomyolipomas. We report a case of a young female with LAM who was admitted to the hospital for a COVID-19 infection, with the objective of discussing the management of LAM with concurrent COVID-19 infection. She ultimately showed overall clinical improvement after receiving dexamethasone and remdesivir, while holding her outpatient mammalian target of rapamycin (mTOR) inhibitor. When patients with rare diseases acquire COVID-19, an individualized approach to treatment is often most effective as information and studies may be limited.

## Introduction

Lymphangioleiomyomatosis (LAM) is the result of abnormal growth of immature smooth-muscle cells resulting in obstructing and restricting lesions that develop into cysts. Although most commonly affecting the lung parenchyma, benign tumors can be found in other organs such as renal angiomyolipomas [1]. LAM is a rare disease that can develop sporadically, in about three to seven individuals per 1 million, or be inherited in individuals with tuberous sclerosis complex (TSC), affecting up to 80% of women with TSC [2]. LAM is most commonly seen in females of reproductive age, as the estrogen production that occurs during this stage of life is seen as a risk factor [3]. Individuals symptomatic of LAM will typically have dyspnea, less commonly cough and hemoptysis, and rarely pleural effusions and recurrent pneumothoraces [4]. Imaging studies often demonstrate characteristic cystic structures, but can also show honeycombing and reticulonodular structures. Pulmonary function tests typically show an obstructive pattern with lung volumes that are normal to increased. The gold standard, and only definitive method for diagnosis, is an open lung biopsy [1].

This particular case report briefly details an effective management strategy that involved treating acute COVID-19 pneumonia while also managing the patient's immunosuppressive therapy while hospitalized. There are no absolute guidelines for individuals with LAM who acquire COVID-19. Not only does this case report add to the literature on LAM and COVID-19 management, but it also emphasizes the importance of having an established LAM specialist to help guide hospital providers who may not be as familiar with a rare disease process. This case also provides prototypical radiographic findings of LAM and superimposed COVID-19.

## Case presentation

A 29-year-old female with a medical history including TSC, LAM, renal angiomyolipomas, seizure disorder, and moderate persistent asthma presented for evaluation of fatigue in the setting of fevers, dyspnea, nausea, and diarrhea, and was subsequently found to be positive for COVID-19. The patient began to have symptoms one week prior to her hospital presentation, at which time she took an outpatient COVID-19 test which was positive. Her symptoms became progressively worse and she noted her oxygen saturation on pulse oximetry to be in the 70% range on room air, which prompted her to contact her primary care physician and ultimately present to the emergency department. She was scheduled to get her first dose of the COVID-19 vaccine on the same day as the hospital presentation.

She was diagnosed with LAM five years prior to this hospitalization after a prior CT chest demonstrated “multiple variable size small cysts throughout both lungs likely representing LAM and multiple renal lesions likely representing renal angiomyolipoma.” Based on her history of tuberous sclerosis, lack of smoking history, and the radiographic findings on her CT scan, she was clinically diagnosed with LAM without lung biopsy.

Since her LAM diagnosis, she has been closely monitored by her pulmonologist. She was found to have significantly elevated vascular endothelial growth factor-D (VEGF-D) level on lab work. Based on this elevation, the presence of renal angiomyolipomas, and the current ongoing MILED trial [5], it was ultimately determined that she should be started on Sirolimus, which is a mammalian target of rapamycin (mTOR) inhibitor. Although her lung function tests had remained stable, she was having an active extrathoracic disease with multiple angiofibromas, shagreen patches, and multiple bilateral renal angiomyolipomas, which necessitated initiation of medical therapy; she was started on Sirolimus at about 24 months prior to this presentation. Her pulmonary status had been stable on Sirolimus and maintenance therapy of budesonide/formoterol before developing COVID-19.

**Figure 1:**
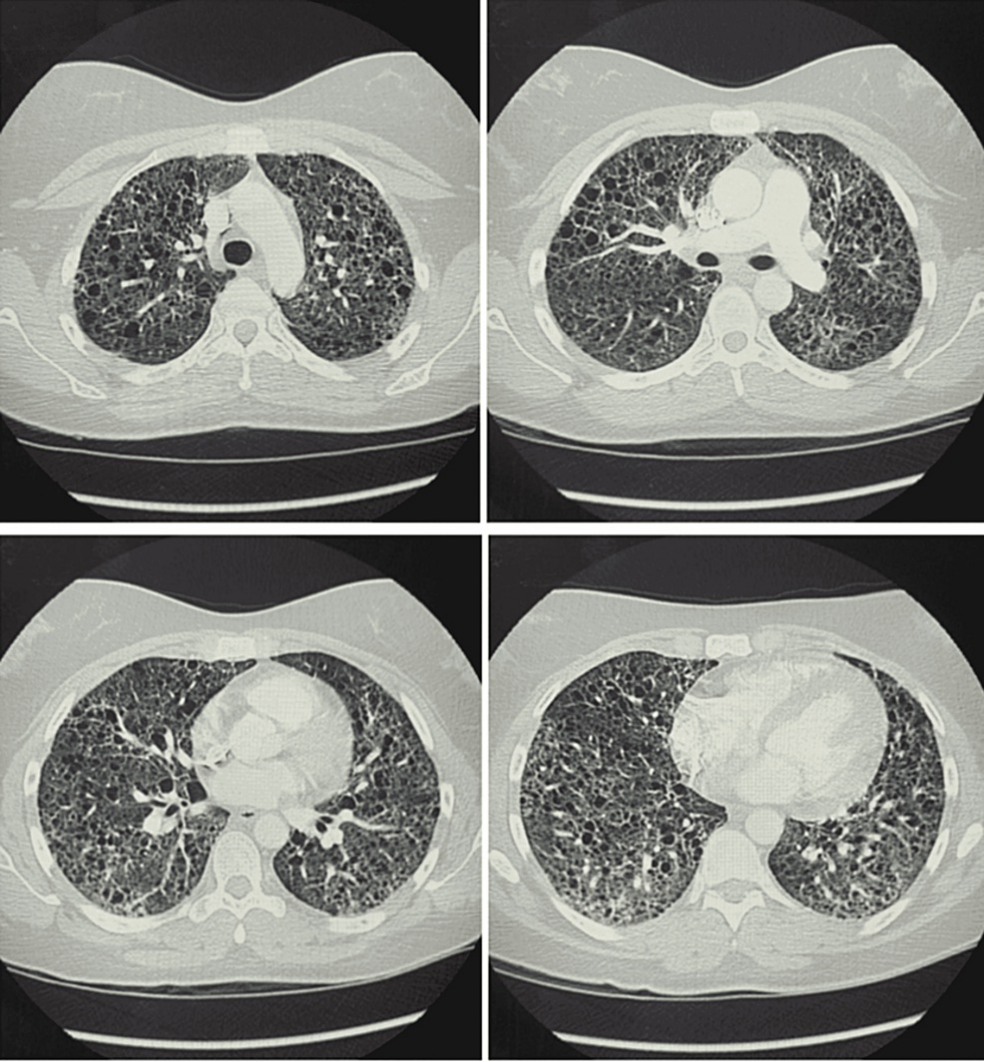
CT chest demonstrating extensive cystic changes throughout the lungs bilaterally and diffuse patchy ground-glass densities, consistent with underlying lymphangioleiomyomatosis and superimposed COVID-19 pneumonia.

During this hospitalization, she underwent a CT chest that demonstrated extensive pulmonary cystic changes as well as diffuse, ground-glass densities, as seen in Figure 1. She required as much as 6 L/min of the nasal cannula to maintain her oxygen saturation on pulse oximetry above 88%. After discussion with her outpatient pulmonologist, her Sirolimus therapy was held in an attempt to limit further immunosuppression while having an acute infection. She was treated with a five-day course of remdesivir per the National Institutes of Health (NIH) COVID-19 guidelines at the time of her hospitalization [6]. She also completed a 10-day course of dexamethasone per the CoDEX trial [7]. She was evaluated for interleukin-6 inhibitor therapy with tocilizumab but was deemed not a candidate with C-reactive protein < 7.50 mg/dL [6].

She gradually showed clinical improvement overall; however, she was unable to be weaned off supplemental oxygen during her hospital stay. She was ultimately discharged home on a 4 L/min nasal cannula after 10 days of hospitalization with instructions to resume Sirolimus and follow up with her pulmonologist.

## Discussion

While the literature on patients with LAM and COVID-19 is limited, patients with LAM may be at an increased risk of contracting infectious diseases, such as COVID-19, due to being on immunosuppressant agents. Sirolimus is FDA approved for LAM and organ transplant rejection and works as an mTOR inhibitor. mTOR signaling regulates multiple cellular functions, including cell growth and lymphangiogenesis, and LAM lesions in the lung exhibit activation of the mTOR pathway. Furthermore, an mTOR inhibitor, such as Sirolimus, blocks inappropriate activation of mTOR and allows restoration of proper cell function [8].

The MILES trial, published in 2011, looked at patients with moderate lung damage in the setting of LAM to assess the utility of Sirolimus therapy. The study demonstrated that the treatment of patients with moderate to severe LAM-related lung disease with sirolimus for one year helped stabilize FEV1, improve FVC, and improve some functional performance measures [8].

The MILED trial, a follow-up to the MILES trial, is currently being conducted through the Rare Lung Disease Clinic Network [5]. This study focused on determining if Sirolimus can be used at low doses in patients with the early disease to prevent progression to more advanced, symptomatic stages. As a result, some physicians have begun to use Sirolimus in patients with preserved lung function in an effort to prevent future lung damage, such as with our patient.

Only a few studies have looked at patients with LAM on mTOR inhibitors to evaluate whether it places patients at greater risk of contracting COVID-19 or if it could have beneficial effects. In a retrospective cohort study by Peron et al. [9], they evaluated 102 Italian individuals with TSC and/or LAM to evaluate the possible effects of mTOR inhibitors on COVID-19 with results suggesting no increased risk of developing COVID-19 as none of the patients on mTOR inhibitors developed confirmed COVID-19 infection [9]. Following the Peron et al. study, Baldi et al. [10], conducted a retrospective study to assess LAM, COVID-19, and the role of mTOR inhibitors in patients from Brazil. Findings demonstrated similar infection rates between patients actively on mTOR inhibitors and those who were not, suggesting that mTOR inhibitors do not increase the risk of symptomatic COVID-19 [10].

The LAM Foundation is a non-profit organization that educates and advocates for those affected by LAM by summarizing current information and raising funds for future research. The LAM foundation strongly recommends against discontinuing Sirolimus therapy in an attempt to decrease the risk of contracting COVID-19. For patients with LAM who develop an active COVID-19 infection, the LAM Foundation recommends discussing reducing or holding Sirolimus therapy with the patient’s LAM physician [11].

## Conclusions

This case study illustrates a patient with underlying LAM and TSC who developed acute hypoxemic respiratory failure secondary to COVID-19. While the literature on patients with LAM and COVID-19 is limited, recommendations should be based on guideline-directed therapy for COVID-19, and management of mTOR inhibitors should be made on an individual basis.
